# Dose-dependent hemato-biochemical and genotoxic responses of common carp (*Cyprinus carpio*) to flupyradifurone

**DOI:** 10.3389/fphys.2025.1676992

**Published:** 2025-10-02

**Authors:** Önder Yıldırım, Ümit Acar, Rifat Tezel, Yavuz Erden, Gökçen Bilge, Sercan Yapıcı

**Affiliations:** ^1^ Faculty of Fisheries, Muğla Sıtkı Koçman University, Muğla, Türkiye; ^2^ Bayramiç Vocational School, Çanakkale Onsekiz Mart University, Çanakkale, Türkiye; ^3^ Faculty of Science, Bartın University, Bartın, Türkiye

**Keywords:** comet assay, common carp, fish haematology, lethal concentration, pesticides

## Abstract

Flupyradifurone (FPF), a systemic butenolide insecticide introduced in 2014, is increasingly used as an alternative to neonicotinoids, yet its safety for non-target aquatic organisms remains poorly understood. This study evaluated the acute and sub-lethal toxicity of FPF in juvenile common carp (*Cyprinus carpio*). A 96-h static bioassay determined an LC_50_ of 140.47 mg/L. Fish were then exposed for 14 days to sub-lethal concentrations (1, 3, 5, 25, 75 and 125 mg/L) to assess hematological, biochemical, and genotoxic responses. Hematological analysis revealed significant, dose-dependent declines in red blood cells (1.71 × 10^6^/μL in control vs. 1.12 × 10^6^/μL at 125 mg/L), hemoglobin (8.34 vs. 3.34 g/dL), and hematocrit (26.08% vs. 13.73%), accompanied by reduced mean corpuscular volume, mean corpuscular hemoglobin, and mean corpuscular hemoglobin concentration at higher doses, indicating anemia and impaired oxygen transport. Biochemically, glucose increased sharply (102.21 mmol/L in control to 230.29 mmol/L at 125 mg/L), while triglycerides, cholesterol, total protein, and albumin declined significantly, suggesting metabolic disruption. Hepatic enzyme activities (alkaline phosphatase, serum glutamic oxaloacetic transaminase, serum glutamate pyruvate transaminase) increased markedly, with serum glutamic oxaloacetic transaminase rising from 36.47 U/L in controls to 144.02 U/L at 125 mg/L, indicative of hepatocellular damage. Comet assay confirmed pronounced DNA damage at ≥25 mg/L, with significant elevations in tail length, tail moment, and % DNA in tail. Collectively, these results demonstrate that FPF exposure compromises hematological health, disrupts metabolic balance, and induces genotoxicity in common carp, even at sub-lethal concentrations. Incorporating both physiological and genomic endpoints is essential for comprehensive ecological risk assessments of emerging insecticides.

## 1 Introduction

Pesticides are essential for managing pests that threaten agricultural production; nonetheless, their extensive use has generated considerable ecological and health issues. Residual pesticides may pollute surface water via runoff, endangering aquatic organisms and infiltrating the human food chain, potentially affecting human health (Campbell et al., 2016). These substances are associated with extensive environmental problems, including soil and water contamination, as well as persistent negative effects on ecosystem structure and function. Pesticide residues can accumulate over time, leading to alterations in biodiversity, disruption of trophic interactions, and impairment of ecosystem services such as nutrient cycling and water purification. Such ongoing disturbances may gradually undermine the resilience and stability of ecosystems. The toxicity of pesticides to non-target species is particularly concerning, since it can disrupt ecological balance and pose risks to food safety (Cheng et al., 2020). Escalating resistance to widely used pesticides, including neonicotinoids, has heightened the desire for novel pest management options (Bass et al., 2015), but they too entail hazards. Due to the interplay of environmental stresses such as climate change, urbanization, and increased agriculture, there is an imperative need for enhanced pesticide risk assessments that include both lethal and sub-lethal impacts on ecosystems and human health (Goulson et al., 2015).

Flupyradifurone (FPF) is a systemic butenolide insecticide launched in 2014 as a substitute for neonicotinoids, especially for controlling neonicotinoid-resistant pests (Nauen et al., 2015). It acts as a nicotinic acetylcholine receptor (nAChR) agonist, similar to neonicotinoids, although with a unique pharmacophore structure (Jeschke et al., 2015). FPF is used on several crops, including tomatoes, grapes, and citrus, to target pests such as aphids, whiteflies, and thrips (Bayer, 2013). Although FPF is often used as a replacement for imidacloprid in agriculture (Goodhue et al., 2020), its long-term ecological effects, especially on aquatic organisms, are inadequately researched (Siviter et al., 2023). Although the concentrations of flupyradifurone reported in natural waters are relatively low, the highest level recorded to date reached 0.16 μg/L in the Great Lakes basin (Metcalfe et al., 2019). However, due to its high solubility and persistence, there is concern that long-term agricultural use may lead to accumulation in freshwater ecosystems. Therefore, understanding both lethal and sublethal effects in fish is crucial to assess the ecological risks of FPF.

Sublethal exposure to pesticides often causes pronounced alterations in fish hematological and biochemical parameters, which are widely recognized as sensitive indicators of physiological stress and health status. Hematological indices such as red blood cell count, hemoglobin concentration, and hematocrit provide insight into oxygen transport and anemia induced by toxicants, while changes in leukocyte counts reflect immunological disturbances (Adhikari et al., 2004; Ramesh and Saravanan, 2009; Öz et al., 2024). Biochemical markers are equally important: elevated plasma glucose is a common response to stress, whereas alterations in total protein and albumin indicate impaired hepatic protein synthesis (Yilmaz et al., 2021). Hepatic enzymes including alanine aminotransferase (ALT), aspartate aminotransferase (AST), and alkaline phosphatase (ALP) are biomarkers of liver dysfunction, and their elevation signals hepatocellular damage under pesticide stress (Oruc and Üner, 1999). In addition, acetylcholinesterase (AChE) inhibition is a well-documented biomarker of neurotoxicity caused by several insecticides (Banaee et al., 2013). Collectively, these hematological and biochemical endpoints provide an integrated picture of how sublethal pesticide exposure disrupts multiple physiological systems in fish, making them highly relevant for assessing the toxic potential of novel insecticides such as FPF.

The common carp (*Cyprinus carpio*), belonging to the Cyprinidae family, is a resilient freshwater fish species native to Eurasia. This species has been extensively spread globally due to their adaptability and durability and it is now occurring in several countries outside its native range. Common carp thrive in a variety of aquatic environments, notably preferring slow-moving water bodies such as rivers with diminished flow, ponds, and lakes. They have a remarkable capacity to tolerate well different environmental conditions (Ji et al., 2012). Their resilience, together with their low maintenance needs and compatibility with many species, have contributed to rend common carp one of the favorite species to be used in scientific research especially those aiming to examine the impact of environmental pollutants on aquatic organisms. Their extensive distribution and accessibility further increase their appropriateness for such trials. In addition to their ecological relevance, common carp also hold substantial commercial value in many regions (Khoshnood, 2024).

Therefore, the objective of this study was to determine the dose-dependent hemato-biochemical alterations and genotoxic responses in common carp (*C. carpio*) exposed to different concentrations of flupyradifurone under controlled laboratory conditions. By establishing median lethal concentration (LC_50_) values and evaluating blood parameters, serum biochemistry, and comet assay results after sublethal exposure, this study aims to provide new insights into the potential ecological risks of this insecticide to freshwater fish species.

## 2 Materials and methods

### 2.1 Fish and experimental conditions

The research protocol received approval from the institutional ethics board (MSKU-HADYEK Local Ethics Commission of Animal Research, Reference number: 2024/10-1). Juvenile common carp (*C. carpio*) were sourced from the Central Fisheries Research Institute in Antalya, Türkiye. The fish were distributed among 50 L glass aquaria at a density of 10 fish per tank in triplicate and given a week to adapt to their new environment and fed twice daily at a rate of 2% their body weight with commercial pellets containing 28% protein and 3.5% lipid (Tetra, Germany).

Flupyradifurone (FPF) was purchased from Sigma-Aldrich (St. Louis, MO, United States) with a reported purity of 99.9% (Lot# BCCB1463). A stock solution was prepared at a concentration of 100 mg/mL using dechlorinated water, given the high-water solubility of the compound (3.2 g/L at 20 °C). The stock solution was stored in amber-colored vials at +4 °C in the dark. Prior to the experiment, it was appropriately diluted with water to achieve the desired exposure concentrations. To establish the LC_50_ value, common carp were first subjected to a preliminary range-finding test to identify appropriate concentration intervals of flupyradifurone. Based on the observed mortality after 96 h in the preliminary test, the definitive assay was conducted in triplicate at concentrations of 0, 1, 3, 5, 25, 75, 125, 175, 225, and 325 mg/L using static aquariums, with 10 fish per aquarium (30 aquariums in total; mean body weight 20.80 ± 0.73 g). Fish were not fed during the exposure period, in accordance with standard acute toxicity testing protocols. Daily mortality was recorded, and the median lethal concentration (LC_50_) and 95% confidence limits were calculated using Probit analysis (Finney, 1952), following the procedure described in AAT Bioquest (2025). During the 96 h-LC_50_ experiment, fish were left to starving. The mortality was checked twice daily, and the dead fish were taken out instantly. Based on the LC_50_ results, concentrations of 0, 1, 3, 5, 25, 75 and 125 mg/L were chosen to evaluate the fish’s physiological reactions to the pesticide. The specimens, with an average weight of 21.77 ± 0.64 g, were exposed to pesticide for a 2-week period. During the chronic exposure period, fish were fed a commercial pelleted diet (40% CP and 12% CL) suitable for *C. carpio* at a rate of approximately 2% of body weight per day. Feeding was conducted once daily, and any uneaten feed was removed to maintain water quality. Exposure solutions were renewed every 48 h with 100% replacement to maintain the stability of pesticide concentrations. Water renewal was conducted carefully to minimize handling stress, and all physicochemical parameters were matched to the previous conditions. This procedure follows semi-static test protocols recommended in OECD guidelines. Each treatment group consisted of three replicate tanks, each containing 10 fish, resulting in 30 fish per treatment concentration. After the chronic exposure period, blood samples were collected from fish sedated with 20 mg/L clove oil. For tissue sampling, fish were humanely euthanized using a higher clove oil concentration (200 mg/L), following the protocol described by Yılmaz and Ergün (2018).

Throughout the experiment water parameters were measured daily and water conditions were carefully regulated to suit carp requirements. The temperature was maintained at 24.68 °C ± 0.14 °C and 24.35 °C ± 0.31 °C for 96 h-LC_50_ and 14-day exposure treatments respectively. The oxygen content was observed as 6.44 ± 0.27 mg/L and 6.39 ± 0.32 mg/L for 96 h-LC_50_ and 14-day exposure treatments respectively.

### 2.2 Hematological and biochemical analysis

Prior to blood collection, the region between the caudal and anal fins was thoroughly sanitized with an alcohol solution to eliminate any potential contamination from mucus. A sterile plastic syringe was then used to extract blood from the caudal vein in this cleansed area. The collected blood was divided into two portions: one was transferred to tubes containing heparin for hematological analysis, while the other was distributed between K3EDTA tubes and gel serum tubes for biochemical and immunological evaluations, respectively.

To obtain serum, the gel tubes were subjected to centrifugation at 4 °C at 3,500 g for a duration of 15 min. Hematological parameters such as in red blood cells (RBC) count, hemoglobin (Hb) levels, and hematocrit (Hct) were evaluated using an automated blood analyzer (Mindray BC3000) that had been specifically calibrated for fish blood samples. The erythrocyte indices such as mean corpuscular volume (MCV), mean corpuscular hemoglobin (MCH), and mean corpuscular hemoglobin concentration (MCHC) were subsequently calculated based on these measurements use formulae below:

$$\text{MCV }\left(\text{fl}\right)=\frac{\text{Hct}\times 10}{\text{RBC}\;\left(10^{6}/\mu L\right)}$$

$$\text{MCH}\left(\text{pg}/\text{cell}\right)=\frac{\text{Hb}\left(g/\text{dL}\right)\times 10}{\text{RBC}\;\left(10^{6}/\mu L\right)}$$

$$\text{MCHC}\left(\%\right)=\frac{\text{Hb}\left(g/\text{dL}\right)\times 100}{\text{Hct}}$$

A range of biochemical markers were assessed using photometric techniques, employing test kits supplied by Bioanalytic Diagnostic Industry Co. These markers included: Glucose (GLU), Triglycerides (TRI), Cholesterol (CHOL), Total protein (TPROT), Albumin (ALB), Alkaline phosphatase (ALP), Serum glutamic oxaloacetic transaminase (SGOT); Serum glutamate pyruvate transaminase (SGPT).

### 2.3 Comet assay

Whole blood sample of 2 μL was mixed with 80 μL of low-melting-point agarose. This mixture was then carefully transferred onto microscope slides previously prepared with a coating of 1% high-melting-point agarose. The slides were covered with coverslips and kept in a dark environment at +4 °C for 10-15 min to allow the agarose to set. After removing the coverslips, the slides were submerged in a prepared lysis solution (2.5 M NaCl, 100 mM EDTA, 10 mM Tris, pH 10). Samples were then transferred to a freshly made working solution, created by adding 1% Triton X-100% and 10% DMSO to the lysis buffer. Following the lysis process, the slides were positioned in a horizontal electrophoresis apparatus (Cleaver Scientific, United Kingdom). The voltage was set to 25 V with a current of 300 mA, and electrophoresis was conducted for 30 min. Post-electrophoresis, the slides underwent three 5-min rinses with a neutralizing buffer (0.4 M Tris, pH 7.5) at +4 °C. For visualization, 50 μL of ethidium bromide solution (20 μg/mL) was applied to each slide, which was then covered with a coverslip. The prepared slides were examined under a fluorescence microscope (Carl Zeiss/Scope A1, Germany). Images of the comet test were taken for analysis and head diameter, tail length, and fluorescence intensity of the samples were calculated in the analysis program of the microscope (Zeiss ZEN blue edition software). At least 25 cells were evaluated for each control and experimental group. DNA damage was determined by measuring head and tail lengths, the ratio of tail length to total DNA length (% DNA tail), and tail moment length (Burlinson, 2012).

### 2.4 Statistical analysis

Data analysis was performed using SPSS 22.0 software (SPSS Inc., Chicago, United States) and reported as mean ± SD and median (min-max). Shapiro-Wilk normality tests and Levene’s Homogeneity test were used to analyze the normality of data and homogeneity of variances, respectively. One-way analysis of variance (ANOVA) was used to determine whether there was variation among treatments in the parameters where assumptions were met. Tukey and Tamhane’s T2 Post-Hoc tests were used to determine differences between groups. In cases where assumptions were not met, multiple comparisons were made with Kruskal–Wallis test, and pairwise comparisons were made with Dunn’s multiple comparisons test. The significance level for each analysis was set at 0.05.

## 3 Results

### 3.1 Acute toxicity

**FIGURE 1:**
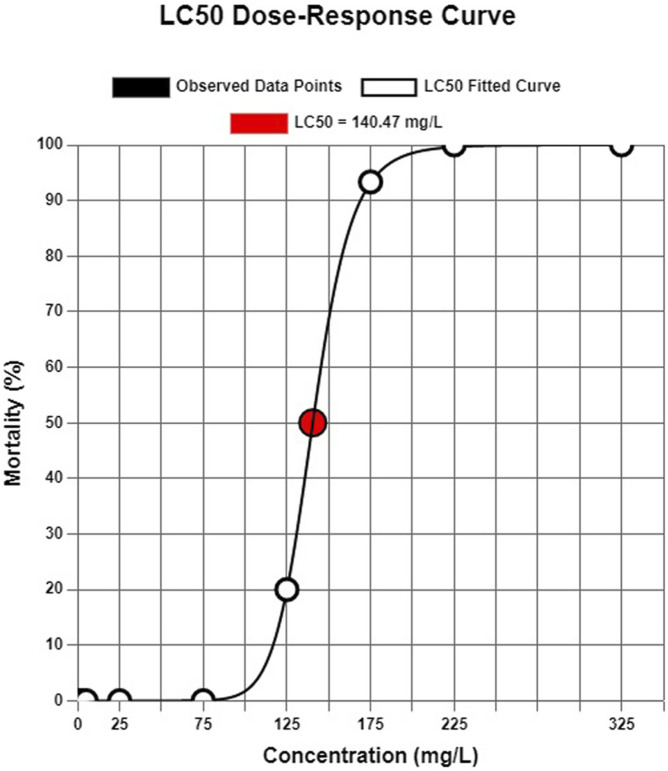
Probit model-based estimation of LC_50_ value for flypiradifurone on common carp (*Cyprinus carpio*).

In the acute toxicity test, it was calculated that flupyradifurone had a 140.47 mg/L of 96-h LC_50_ for common carp (Figure 1). No mortality was observed at the sub-lethal concentrations of flupyradifurone and negative control (0.01% DMSO) during the 14 days.

### 3.2 Hematological and serum biochemical parameters

The changes in hematological parameters are summarized in Table 1. Increasing concentrations of the tested compound resulted in a significant reduction in RBC, Hb levels and Hct values across the experimental groups. In addition, dose-dependent alterations were observed in MCV, MCH, and MCHC. MCV, MCH and MCHC levels decreased significantly at higher doses. These findings indicate that the hematological parameters were adversely affected by escalating doses compared to the control group. Specifically, RBC, Hb, and Hct values declined significantly in a dose-dependent manner, indicating anemia and impaired oxygen transport, while erythrocyte indices (MCV, MCH, MCHC) were also markedly reduced at 75–125 mg/L.

**TABLE 1:** Effects of different concentrations of flupyradifurone on the hematological parameters of common carp (*Cyprinus carpio*) after 14 days of exposure.

Parameters Groups	RBC (x10^6^/μL)	Hb (g/dL)	Hct (%)	MCV (fL)	MCH (pg/cell)	MCHC (%)
Control	1.71 ± 0.09^d^	8.34 ± 0.42^d^	26.08 ± 1.30^d^	152.24 ± 3.53^bc^	32.02 ± 1.11^c^	48.74 ± 2.11^bc^
1 mg/L	1.70 ± 0.04^d^	8.21 ± 0.35^d^	25.33 ± 0.83^d^	149.32 ± 3.17^bc^	32.41 ± 0.86^c^	48.39 ± 1.60^bc^
3 mg/L	1.69 ± 0.06^d^	8.56 ± 0.25^d^	24.76 ± 0.49^d^	146.47 ± 2.66^c^	34.56 ± 0.94^d^	50.62 ± 1.54^c^
5 mg/L	1.,33 ± 0.05^c^	6.52 ± 0.24^c^	20.74 ± 0.57^c^	155.57 ± 5.18^b^	31.45 ± 1.21^bc^	48.90 ± 1.51^bc^
25 mg/L	1.31 ± 0.04^bc^	6.16 ± 0.19^c^	20.46 ± 0.32^c^	155.71 ± 4.40^b^	30.11 ± 1.26^b^	46.86 ± 1.86^b^
75 mg/L	1.25 ± 0.05^ab^	3.77 ± 0.23^b^	16.63 ± 0.43^b^	133.44 ± 4.85^a^	22.64 ± 1.12^a^	30.21 ± 1.93^a^
125 mg/L	1.12 ± 0.09^a^	3.34 ± 0.21^a^	13.73 ± 0.38^a^	123.23 ± 12.01^a^	24.38 ± 1.81^a^	29.93 ± 2.35^a^
P value	<0.001	<0.001	<0.001	<0.001	<0.001	<0.001

The values (n = 15) are mean ± standard deviation values with different letters in the same column indicate significant differences between the groups (P < 0.05). Note: RBC (red blood cells); Hb (haemoglobin concentration); Hct (haematocrit); MCV (mean corpuscular volume); MCH (mean corpuscular haemoglobin); MCHC (mean corpuscular haemoglobin concentration).

In this study, significant and dose-dependent effects of flupyradifurone exposure on the biochemical parameters of fish were observed. GLU levels showed a significant increase at concentrations of 25 mg/L and above compared to the control group. In contrast, TRIG and CHOL levels decreased as concentration increased, reaching their lowest values, particularly in groups exposed to 75 mg/L and higher. Liver function markers, including ALP, SGOT, and SGPT activities, exhibited notable increases (Table 2). Compared to the control group, SGOT activity increased approximately fourfold (P < 0.05) and SGPT activity nearly twofold (P < 0.05) in the 125 mg/L group. Additionally, TPROT (total protein) and ALB (albumin) levels showed a dose-dependent decrease with increasing flupyradifurone exposure (P < 0.05).

**TABLE 2:** Effects of different concentrations of flupyradifurone on the serum biochemical parameters of common carp (*Cyprinus carpio*) after 14 days of exposure.

Groups Parameters	Control	1 mg/L	3 mg/L	5 mg/L	25 mg/L	75 mg/L	125 mg/L	P value
GLU (mmol/L)	102.21 ± 15.05^a^	95.11 ± 12.17^a^	95.76 ± 7.47^a^	115.76 ± 14.26^a^	179.39 ± 4.11^b^	180.29 ± 10.52^b^	230.29 ± 9.81^c^	<0.001
TRIG (mmol/L)	74.14 ± 6.20^c^	69.68 ± 8.88^c^	65.22 ± 10.88^bc^	64.91 ± 6.29^c^	52.02 ± 4.50^b^	39.09 ± 5.20^a^	33.27 ± 6.98^a^	<0.001
CHOL (mmol/L)	159.41 ± 21.64^c^	160.02 ± 10.37^c^	155.05 ± 11.91^c^	150.90 ± 21.22^c^	142.09 ± 12.79^c^	96.28 ± 8.79^b^	79.02 ± 9.18^a^	<0.001
TPROT (g/L)	7.16 ± 0.54^c^	6.93 ± 0.48^c^	7.32 ± 0.49^c^	7.18 ± 0.64^c^	6.73 ± 0.53^c^	5.77 ± 0.31^b^	4.53 ± 0.39^a^	<0.001
ALB	3.72 ± 0.42^d^	3.65 ± 0.24^d^	3.46 ± 0.56^cd^	2.71 ± 0.20^bc^	2.40 ± 0.22^b^	1.84 ± 0.19^a^	1.63 ± 0.20^a^	<0.001
ALP (U/L)	17.27 ± 2.58^a^	17.62 ± 3.14^a^	19.73 ± 1.89^a^	19.29 ± 1.77^a^	29.85 ± 2.69^b^	42.79 ± 4.12^c^	53.18 ± 4.55^d^	<0.001
SGOT (U/L)	36.47 ± 5.91^a^	40.20 ± 4.43^a^	48.75 ± 8.87^ab^	48.52 ± 9.10^ab^	57.62 ± 5.60^b^	128.42 ± 4.43^c^	144.02 ± 13.03^c^	<0.001
SGPT (U/L)	3.72 ± 0.48^a^	3.85 ± 1.37^a^	3.87 ± 0.79^a^	3.98 ± 1.13^a^	4.89 ± 1.24^ab^	6.01 ± 0.66^bc^	8.19 ± 2.00^c^	<0.001

The values (n = 15) with different letters in the same row indicate significant differences between the groups (P < 0.05). Note: GLU (glucose); TRIG (triglycerides); CHOL (cholesterol); TPROT (total protein); ALB (albumin); ALP (alkaline phosphatase); SGOT (serum glutamic oxaloacetic transaminase); SGPT (Serum glutamate pyruvate transaminase).

### 3.3 DNA damage assessment by comet assay

**FIGURE 2:**
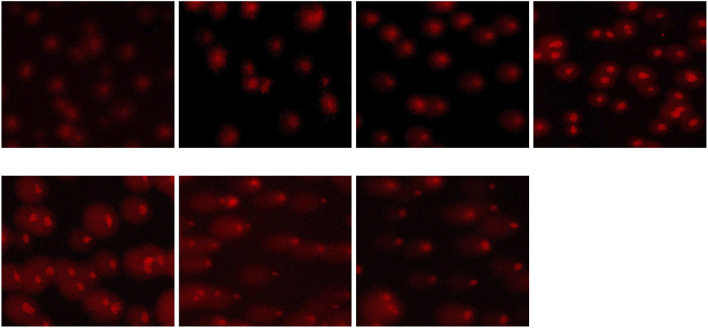
Comet Analysis Images of Increased DNA Damage in Common Carp Blood Cells Exposed to Different Concentrations of Flupyradifurone (Control, 1 mg/L, 3 mg/L, 5 mg/L, 25 mg/L, 75 mg/L, 125 mg/L Flupyradifurone).

The exposition to different concentrations of the pesticide flupyradifurone induces DNA damage in common carp erythrocytes. Microscopic images obtained using the comet assay technique clearly show the concentration-dependent genotoxic effects of flupyradifurone exposure (Figure 2). While the cells in the control group remain compact and intact, increasing pesticide concentrations lead to a noticeable increase in DNA migration, with “comet-like” formations becoming particularly prominent at high doses (≥25 mg/L).

**FIGURE 3:**
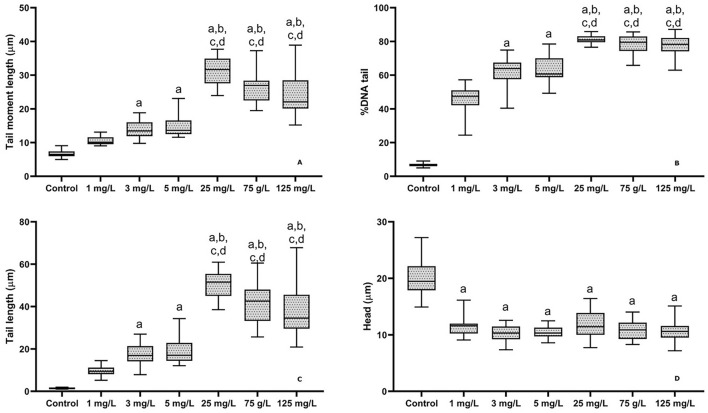
Genotoxic effect of flupyradifurone exposure in different doses common carp blood DNA after 14  days. Tail moment length, % tail DNA, Tail length and head. Results were expressed as median (min-max). Letters on the graph indicate significance (p < 0.05). compared to control, compared to 1 mg/L group, compared to 3 mg/L group, compared to 5 mg/L.

The degree of DNA damage increased significantly with rising flupyradifurone concentrations. Tail length was nearly zero in the control group but showed a significant increase at 25 mg/L, 75 mg/L, and 125 mg/L, with the highest damage observed at approximately 55 μm in the 25 mg/L group (Figure 3). Head size decreased in all exposed groups compared to the control, dropping from approximately 20 μm to around 10-12 μm (Figure 3). Tail moment length followed a similar pattern, showing minimal values in the control group but significant increases at higher concentrations (25, 75, and 125 mg/L) (Figure 3). Notably, the percentage of DNA in the tail exhibited the most dramatic increase, rising from around 5% in the control to 40%-50% at 1 mg/L and reaching 75%-80% at the highest concentrations (Figure 3).

## 4 Discussion

The widespread use of agrochemicals, including novel insecticides such as flupyradifurone, has raised significant concerns regarding their environmental and ecological impacts, particularly in aquatic ecosystems. Flupyradifurone, a butenolide insecticide that targets nicotinic acetylcholine receptors (nAChRs), has gained substantial market interest due to its novel bioactive pharmacophore. However, its potential toxicity in aquatic organisms, including economically and ecologically important fish species like common carp, remains underexplored. In this study, we investigated the toxicological effects of flupyradifurone on common carp to better understand its implications for fish health including hematological, serum biochemical parameters and genotoxicity of blood. Acute toxicity assessment provides a critical step in understanding the environmental risks of emerging pollutants by providing rapid and reliable prediction of their toxic effects. It also allows the determination of safe concentration ranges and thresholds for the study of sublethal effects, providing a basis for long-term ecotoxicological studies (Majumder and Kaviraj, 2019).

According to our findings, the 96-hour LC_50_ of flupyradifurone in common carp was determined to be 140.47 mg/L, which aligns with previously reported values for this species. U.S. Environmental Protection Agency (2014) documented LC_50_ values for common carp as >100 mg/L, consistent with our results and highlighting the moderate acute toxicity of flupyradifurone. Similarly, for other fish species, EFSA reported LC_50_ values of >74.2 mg/L for rainbow trout (*Oncorhynchus mykiss*) and >70.5 mg/L for fathead minnow (*Pimephales promelas*). Although Zhong et al. (2021) found a much lower 96-h LC_50_ value of 0.21 mg/mL for zebra fish embryos (*D. rerio*) also in the same study revealing that flupyradifurone is toxic to zebra fish embryos (*Danio rerio*), resulting in diminished heart rate, survival, and body length, alongside cardiac abnormalities and heightened oxidative stress. Notably, these adverse effects were observed even at relatively low concentrations. This discrepancy underscores the species-specific sensitivity to flupyradifurone and reinforces the importance of targeted toxicological evaluations for ecologically and economically significant fish species like common carp. Although the concentrations applied in this study (1–125 mg/L) are much higher than the currently reported environmental concentrations (up to 0.16 μg/L; Metcalfe et al., 2019), the observed hematological, biochemical, and genotoxic alterations provide valuable insight into potential risks. Importantly, the marked effects at lower sublethal concentrations (1–5 mg/L) suggest that even small increases above current environmental levels could pose ecological hazards, particularly under scenarios of pesticide accumulation and chronic exposure (Metcalfe et al., 2019). Given the physicochemical properties of FPF—namely, high water solubility, low volatility, and persistence in aquatic systems (Nauen et al., 2015) also in aqueous systems under both natural sunlight and artificial light, with hydrolysis half-lives exceeding 150 days at 15 °C, 25 °C, and 35 °C, and photolysis half-lives over 168 h in natural waters (Fang et al., 2022). There is a realistic potential for accumulation in freshwater environments via runoff, erosion, and leaching. Our findings, especially the observed hematological and genotoxic alterations in *C. carpio*, underscore the ecological risks associated with potential long-term and low-dose exposure scenarios.

Hemato-biochemical parameters are essential indicators for assessing fish health and understanding the impact of toxic substances and pollutants and general stress responses observed in fish exposed to various toxicants, where such exposures can lead to physiological adjustments and stress (Kumar et al., 2018; Öz et al., 2020; Çelik et al., 2024). Blood plays a critical role in transporting essential nutrients, gases, hormones, and waste products, making it a reliable source of information on the physiological and biochemical status of fish (Seibel et al., 2021; Ramya et al., 2023). The hematological parameters such RBC, Hct and Hb of common carp were decreased in the present study by flupyradifurone exposure at level of 5, 25, 75 and 125 mg/L meanwhile 1 and 3 mg/L did not show any differences compared to the control group. *Labeo rohita* exposed to sub-lethal concentrations of carbofuran (0.16–0.80 mg/mL) and cypermethrin (0.16–0.80 mg/mL) exhibited a significant, time- and dose-dependent decrease in red blood cell count and hemoglobin levels over a 28-day exposure period (Adhikari et al., 2004). Similar hematological effects have been observed in grass carp (*Ctenopharyngodon idella*) exposed to profenofos-based insecticides (El-bouhy et al., 2023) and in common carp, *L. rohita* and Nile tilapia (*Oreochromis niloticus*) exposed to esfenvalerate, azadirachtin, triflumezopyrim, and hexaflumuron, respectively (Murussi et al., 2016; Navruz et al., 2023; Ibrahim et al., 2024; Nayak et al., 2024). These results emphasize the consistent adverse effects of various insecticides on the hematological health of fish across different species. In this context, the observed decrease in RBC, Hb, and Hct levels in fish exposed to insecticides may be attributed to the destructive effects of these chemicals on cell membranes. According to Mostakim et al. (2015) exposure to toxicants can suppress RBC and Hb synthesis, resulting in reduced RBC counts and Hb levels. Additionally, another potential cause of anemia is the generation of reactive oxygen species (ROS) induced by insecticide exposure, which can damage RBCs and oxidize Hb molecules, thereby impairing their oxygen carrying capacity (Lutnicka et al., 2016). Furthermore, the reductions in RBC, Hb, and Hct levels, which are strongly associated with hematological parameters, are presumed to result from the inhibition of erythropoiesis, destruction of red blood cells, and damage to hematopoietic tissues in the kidney and spleen, and disruption of the hematopoietic process (Korkmaz et al., 2023).

Serum biochemical parameters are essential markers for assessing the toxic effects in fish, with changes in these values often reflecting metabolic imbalances or organ damage (Ma et al., 2018). In vertebrates, glucose (GLU) is the primary energy source, and any surplus is stored as glycogen in the liver and muscles. However, various stressors can alter serum GLU levels, potentially indicating metabolic disturbances or damage to vital organs (Yki-Järvinen, 1992). In our study, exposure to flupyradifurone led to a significant increase in serum GLU levels in common carp. Similar elevations in glucose have been observed in common carp exposed to imidacloprid and chlorpyrifos (Banaee et al., 2024). It is likely that under flupyradifurone induced stress, glycogen stored in the liver and muscles is mobilized via glycogenesis, thereby meeting the heightened energy demands and resulting in increased serum glucose levels. Studies on other contaminants like endosulfan have also reported significant changes in biochemical parameters, including stress biomarker enzymes and blood glucose levels, indicating a general response to toxic exposure (Kumar et al., 2016). Under stress conditions, TRIG plays a role in storing cellular energy and compensating for increased energy demands (Zhu et al., 2024). In the present study, serum TRIG, CHOL, and TPROT levels significantly decreased with increasing doses of flupyradifurone. This reduction in TRIG may result from fish exposed to flupyradifurone attempting to meet their heightened energy needs by performing gluconeogenesis from non-glycogen sources (Zhu et al., 2024). Cholesterol is primarily synthesized in the liver and serves as a crucial structural component of bile acids, plasma lipoproteins, and cell membranes, as well as acting as a precursor for the synthesis of all steroid hormones (Whalan, 2015). Therefore, the decreased CHOL levels may indicate impaired hepatic cholesterol synthesis (Yki-Järvinen, 1992). Meanwhile, reduced TPROT may be associated with compromised immune function and decreased resistance to diseases (Banaee et al., 2024). Similar findings were reported by Zhu et al. (2015) in common carp following fenpropathrin exposure. Furthermore, significant reductions in total serum protein were observed in *L. rohita* juveniles (Whalan, 2015) and *C. catla* juveniles (Simons and Ikonen, 2000) exposed to sub-lethal cypermethrin levels. Likewise, decreases in triglycerides and cholesterol were observed in *O. mykiss* (Orun et al., 2014) and *Schizothorax esocinus* (Akhtar et al., 2021) following cypermethrin exposure.

This study demonstrated that flupyradifurone exerts dose-dependent genotoxic effects on common carp blood DNA, indicating that this pesticide poses a potential hazard to aquatic organisms. While such genotoxic effects may be partially repaired by cellular mechanisms, repeated or chronic exposure could surpass repair capacity, leading to cumulative genetic alterations (Livingstone, 2001). Although individual fish may recover to some extent, persistent DNA damage could impair growth, reproductive success, and survival, potentially affecting population dynamics over time (Galloway and Goven, 2006). These findings underscore the importance of assessing long-term and multigenerational effects of Flupyradifurone to better understand its ecological risks. One primary mechanism of pesticide-induced DNA damage is oxidative stress, whereby exposure elevates reactive oxygen species (ROS) production in fish tissues, which in turn attack DNA molecules and cause strand breaks and base modifications (Das and Mukherjee, 2003; Tunçsoy, 2021). For example, cypermethrin exposure in *L. rohita* produced marked increases in ROS levels and lipid peroxidation in gill, muscle, brain, and liver tissues effects that were effectively mitigated by vitamin C supplementation (Das and Mukherjee, 2003). Another key mechanism involves the formation of DNA adducts certain pesticides or their metabolites can covalently bind to nucleophilic sites on DNA, disrupting its structure and impairing replication and transcription (Vani et al., 2012). Khisroon et al. (2021) showed that endosulfan exposure in *C. idella* not only elevated ROS production but also induced DNA adduct formation in a time and dose-dependent manner. Interestingly, the comet assay revealed a sharp increase in DNA damage even at the lowest tested concentration (1 mg/L), with tail DNA reaching 40%–50%. Such an early and pronounced response may be attributed to the high sensitivity of erythrocyte DNA to oxidative stress induced by flupyradifurone. Similar non-linear or threshold-type responses have been reported in fish exposed to other pesticides, where low doses were sufficient to trigger substantial genotoxic effects before dose-dependent increases were observed (Sharma and Jindal, 2022). Another possible explanation is inter-individual variability in DNA repair capacity, which can lead to disproportionate damage at lower concentrations. Although methodological factors inherent to the comet assay may also contribute to variability, the consistent trend of increasing damage with higher concentrations supports the biological relevance of this observation. Chromosomal aberrations have likewise been observed: acute exposure to the glyphosate-based herbicide Templo® induced erythrocyte morphological alterations and micronucleus formation in yellowtail tetra (Ullah et al., 2024), highlighting the clastogenic and cytotoxic potential of such formulations. Moreover, organophosphate insecticides can disrupt enzymatic processes that contribute to DNA damage; Alavinia et al. (2019) found that malathion exposure in *O. mykiss* caused DNA strand breaks concomitant with alterations in acetylcholinesterase activity, indicating genotoxic effects beyond neurotoxicity. The random amplified polymorphic DNA (RAPD) assay has also proven sensitive in detecting pesticide-induced genetic alterations: Pandey et al. (2018) documented RAPD profile changes in *Channa punctatus* following profenofos exposure, reflecting underlying DNA damage. Finally, the bioaccumulation of persistent pesticides in aquatic ecosystems can amplify genotoxic risk through trophic transfer and tissue accumulation (Ergenler and Turan, 2023).

## 5 Conclusion

In conclusion, the present study demonstrates that flupyradifurone exerts significant dose-dependent toxic effects on common carp, as evidenced by its moderate acute toxicity (96 h-LC_50_ = 140.47 mg/L) and clear sub-lethal impacts over 14 days of exposure. Hematological analyses revealed marked declines in RBC count, hemoglobin concentration, and hematocrit, alongside alterations in erythrocyte indices (MCV, MCH, MCHC), indicating anemia and impaired oxygen transport. Biochemical profiling showed elevated glucose and hepatic enzyme activities (ALP, SGOT, SGPT), together with reductions in triglycerides, cholesterol, total protein, and albumin, reflecting metabolic stress and potential liver dysfunction. The comet assay further confirmed pronounced genotoxicity, with significant increases in tail length, tail moment, and percentage tail DNA at concentrations ≥25 mg/L. Collectively, these findings underscore the ecological risk posed by flupyradifurone to freshwater fish and highlight the importance of incorporating both lethal and sub-lethal endpoints—including genotoxic and biochemical biomarkers—into pesticide risk assessments. Future work should extend to chronic exposures and trophic-transfer studies to fully elucidate long-term environmental implications.

## Data Availability

The original contributions presented in the study are included in the article/Supplementary Material, further inquiries can be directed to the corresponding author.
