# Supplementary material for: Dose-dependent hemato-biochemical and genotoxic responses of common carp (Cyprinus carpio) to flupyradifurone
Source: Front Physiol. 2025 Oct 2;16:1676992. doi: 10.3389/fphys.2025.1676992 (PMC12528199; doi:10.3389/fphys.2025.1676992)
Supplement: Supplementary file 1 [file DataSheet1.zip › Peerj_Raw_Datas/%DNA tail data.pdf]

	Group A	Group B	Group C	Group D	Group E	Group F	Group G
	Control	1 mg/L	3 mg/L	5 mg/L	25 mg/L	75 g/L	125 mg/L
1	6.490000	37.58639	55.79138	61.39288	81.94986	78.03166	73.34737
2	6.106667	52.67053	58.15458	53.45404	79.60810	77.87588	78.94511
3	6.376667	51.84360	70.99237	58.87988	82.87508	84.63462	78.92730
4	6.686667	55.42376	63.93865	63.67435	80.54166	81.58548	76.31254
5	5.820000	48.63168	62.19386	74.39653	80.71556	82.91877	78.16902
6	8.096667	47.18182	65.93640	78.46374	78.72040	82.85502	76.92959
7	7.856667	30.16878	67.99393	60.65336	79.65968	83.10391	74.48781
8	6.596667	47.86236	71.02489	74.11560	76.57200	79.51736	78.25875
9	5.603333	44.40502	64.84700	63.20358	79.46098	85.64000	85.66967
10	6.976667	48.15182	61.17095	68.49014	81.85044	79.81527	81.91952
11	5.960000	47.29901	50.49276	58.62441	80.87187	81.18947	87.13085
12	6.403333	50.67168	56.99867	65.87780	82.06731	83.04372	83.90360
13	6.893333	51.25024	63.93542	58.27212	84.45276	84.65230	84.37868
14	4.973333	47.48299	60.30732	60.62097	77.98525	77.91484	84.93978
15	8.066667	41.84474	64.67800	60.67151	80.35850	82.06666	82.23543
16	6.290000	49.57004	60.24010	58.27863	83.49756	77.45682	74.14496
17	7.526667	42.36592	50.63387	60.09585	85.83195	85.49781	74.05729
18	9.070000	43.23725	66.17460	49.26332	83.28510	65.78023	62.95097
19	6.750000	43.28358	56.84596	51.06739	80.87706	73.83938	79.45588
20	5.926667	49.69886	74.86052	72.67219	78.59005	69.34154	77.06416
21	7.400000	57.21477	71.68038	65.09481	81.50374	76.60082	71.96934
22	5.723333	40.59582	67.15576	71.52646	85.36789	68.86052	79.75651
23	6.226667	51.29229	67.75721	59.78734	84.08651	74.27036	68.67090
24	7.363333	41.33612	40.38860	59.70614	82.90470	74.37021	79.01179
25	5.960000	24.43195	61.49449	74.29128	77.92197	73.41909	72.01889
