# Supplementary material for: Dose-dependent hemato-biochemical and genotoxic responses of common carp (Cyprinus carpio) to flupyradifurone
Source: Front Physiol. 2025 Oct 2;16:1676992. doi: 10.3389/fphys.2025.1676992 (PMC12528199; doi:10.3389/fphys.2025.1676992)
Supplement: Supplementary file 1 [file DataSheet1.zip › Peerj_Raw_Datas/Flupyradifurone Blood Parameters Raw Data.pdf]

Group	Hb	Hct	RBC	MCV	MCHC	MCH	Trigliserid	Cholesterol	Albumin	Glucose	TotalProtei	ALP	SGOT	SGPT
1,00	7,50	23,40	1,55	150,97	48,39	32,05	81,77	135,83	2,95	77,20	6,54	17,92	43,57	4,35
1,00	8,80	27,10	1,81	149,72	48,62	32,47	78,15	149,00	4,22	97,91	6,51	14,83	32,34	3,41
1,00	8,70	26,10	1,69	154,44	51,48	33,33	68,92	152,90	3,82	77,20	6,66	19,92	36,32	2,86
1,00	8,50	26,20	1,65	158,79	51,52	32,44	83,08	165,33	3,34	106,69	7,00	14,75	30,88	2,37
1,00	8,80	26,90	1,76	152,84	50,00	32,71	69,23	162,16	3,60	112,97	7,02	20,92	43,42	4,24
1,00	8,20	25,10	1,65	152,12	49,70	32,67	78,46	133,51	3,64	115,48	7,52	16,83	45,07	4,93
1,00	8,10	25,30	1,73	146,24	46,82	32,02	66,92	150,58	3,66	112,97	7,91	19,58	33,39	3,80
1,00	8,30	27,10	1,80	150,56	46,11	30,63	69,23	196,60	3,86	110,34	7,84	13,42	32,40	3,10
1,00	8,20	27,50	1,78	154,49	46,07	29,82	71,54	188,80	4,35	109,13	7,45	17,25	30,80	4,42
2,00	7,80	24,30	1,67	145,51	46,71	32,10	66,62	145,21	3,34	80,96	6,68	12,42	35,34	3,96
2,00	8,60	26,50	1,72	154,07	50,00	32,45	83,08	152,47	3,55	82,85	6,45	18,17	40,51	2,44
2,00	8,40	25,00	1,67	149,70	50,30	33,60	62,00	152,90	3,90	109,83	6,70	19,92	43,94	3,41
2,00	8,20	25,30	1,70	148,82	48,24	32,41	62,31	155,21	3,60	97,91	7,03	17,08	47,57	5,74
2,00	7,90	24,50	1,69	144,97	46,75	32,24	83,08	158,69	4,13	88,10	7,46	19,92	35,85	5,16
2,00	8,70	26,10	1,78	146,63	48,88	33,33	59,69	164,48	3,60	102,30	6,65	16,67	34,76	5,75
2,00	8,50	26,30	1,72	152,91	49,42	32,32	64,62	161,00	3,66	81,65	6,57	13,95	42,60	2,50
2,00	7,80	25,50	1,70	150,00	45,88	30,59	71,54	179,11	3,41	99,79	6,89	17,67	38,32	2,71
2,00	8,00	24,50	1,62	151,23	49,38	32,65	74,15	171,12	3,64	112,62	7,91	22,75	42,87	2,98
3,00	8,90	24,80	1,70	145,88	52,35	35,89	77,46	152,90	4,03	94,77	7,06	20,67	63,52	3,41
3,00	9,00	25,30	1,78	142,13	50,56	35,57	55,69	154,36	3,38	102,30	7,34	22,50	58,83	4,54
3,00	8,50	24,40	1,63	149,69	52,15	34,84	53,08	148,26	2,82	91,00	6,92	20,97	38,65	3,03
3,00	8,60	25,10	1,75	143,43	49,14	34,26	60,00	158,69	2,73	88,98	7,59	17,00	43,34	3,30
3,00	8,40	24,70	1,68	147,02	50,00	34,01	80,00	167,64	2,99	110,11	7,43	18,83	48,69	4,35
3,00	8,30	23,80	1,58	150,63	52,53	34,87	71,54	158,69	4,27	89,75	8,29	19,92	57,19	5,46
3,00	8,60	24,70	1,69	146,15	50,89	34,82	55,38	173,44	3,21	89,12	7,66	21,25	41,63	3,99
3,00	8,40	24,60	1,68	146,43	50,00	34,15	76,15	149,42	3,73	102,30	6,96	19,50	43,30	3,26
3,00	8,30	25,40	1,73	146,82	47,98	32,68	57,69	132,05	3,95	93,51	6,65	16,92	43,57	3,45
4,00	6,70	20,10	1,33	151,13	50,38	33,33	70,54	149,42	2,97	93,97	8,08	18,92	48,63	4,15
4,00	6,60	20,70	1,28	161,72	51,56	31,88	67,92	135,52	2,86	106,52	6,81	18,17	50,05	3,49
4,00	6,90	21,10	1,45	145,52	47,59	32,70	71,54	130,89	2,81	114,05	6,78	18,75	38,80	4,46
4,00	6,80	21,30	1,36	156,62	50,00	31,92	57,69	126,25	2,84	131,63	6,53	16,80	60,49	4,62
4,00	6,30	20,60	1,30	158,46	48,46	30,58	52,77	189,23	2,47	119,07	6,29	21,58	64,72	4,62
4,00	6,50	20,80	1,32	157,58	49,24	31,25	62,31	136,68	2,77	99,71	7,04	22,00	40,08	5,92
4,00	6,30	21,50	1,34	160,45	47,01	29,30	64,62	163,32	2,34	112,80	7,54	19,92	49,82	3,71
4,00	6,40	20,90	1,33	157,14	48,12	30,62	69,23	173,86	2,69	130,57	7,52	20,08	43,38	2,06
4,00	6,20	19,70	1,30	151,54	47,69	31,47	67,54	152,90	2,64	133,51	7,99	17,42	40,70	2,79
5,00	6,30	20,70	1,34	154,48	47,01	30,43	51,08	123,51	2,76	178,07	6,64	34,23	53,85	5,39
5,00	5,90	20,60	1,36	151,47	43,38	28,64	48,46	116,99	2,25	171,17	5,95	28,50	59,84	6,63
5,00	5,80	21,10	1,30	162,31	44,62	27,49	53,08	142,47	2,60	176,81	6,18	34,25	56,84	4,97
5,00	6,20	20,30	1,28	158,59	48,44	30,54	50,77	149,11	2,12	178,07	6,71	30,33	59,25	5,63
5,00	6,10	20,20	1,30	155,38	46,92	30,20	55,38	152,90	2,47	179,95	7,59	29,75	65,18	3,10
5,00	6,20	20,10	1,26	159,52	49,21	30,85	57,69	148,26	2,56	183,09	6,85	27,00	58,51	4,62
5,00	6,30	20,60	1,30	158,46	48,46	30,58	43,54	149,58	2,44	181,21	7,22	28,17	64,37	3,18
5,00	6,40	20,20	1,36	148,53	47,06	31,68	57,38	149,42	2,22	185,60	7,13	27,50	53,54	4,23
5,00	6,20	20,30	1,33	152,63	46,62	30,54	50,77	146,58	2,17	180,53	6,29	28,92	47,22	6,26
6,00	4,30	17,30	1,25	138,40	34,40	24,86	37,62	97,30	1,82	176,81	5,83	43,25	126,11	4,97
6,00	3,70	16,10	1,17	137,61	31,62	22,98	40,54	105,41	2,11	176,77	5,37	50,25	127,77	6,56
6,00	3,90	16,70	1,35	123,70	28,89	23,35	29,38	97,30	1,73	174,30	6,32	36,75	126,31	6,38
6,00	3,60	16,30	1,26	129,37	28,57	22,09	43,15	96,14	96,14	96,14	96,14	40,75	132,94	6,56
6,00	3,80	16,80	1,23	136,59	30,89	22,62	43,85	104,25	1,69	195,64	5,94	43,42	121,06	5,78
6,00	3,70	16,20	1,25	129,60	29,60	22,84	34,62	103,88	1,99	179,95	5,41	44,17	136,36	6,29
6,00	3,80	17,10	1,27	134,65	29,92	22,22	39,23	97,30	1,52	198,78	5,74	41,58	126,37	5,74
6,00	3,60	16,30	1,20	135,83	30,00	22,09	46,46	86,14	2,07	174,93	5,59	38,25	128,16	6,76
6,00	3,50	16,90	1,25	135,20	28,00	20,71	36,92	78,76	1,86	165,52	5,69	46,67	130,66	5,04
7,00	3,20	13,50	0,98	137,76	32,65	23,70	25,08	73,97	1,44	237,69	4,07	50,00	124,20	8,54
7,00	3,10	14,20	1,02	139,22	30,39	21,83	30,00	82,24	1,69	230,16	4,53	57,92	140,78	6,44
7,00	3,00	13,80	1,20	115,00	25,00	21,74	41,54	70,23	1,47	227,65	4,59	53,00	160,30	6,83
7,00	3,30	14,30	1,04	137,50	31,73	23,08	20,77	92,66	2,02	225,14	5,23	46,25	136,65	8,94
7,00	3,40	13,20	1,20	110,00	28,33	25,76	36,92	84,56	1,78	211,92	4,07	53,25	156,09	7,76
7,00	3,60	13,70	1,22	112,30	29,51	26,28	39,23	91,51	1,60	230,79	4,64	58,25	146,90	6,11
7,00	3,40	13,30	1,14	116,67	29,82	25,56	34,62	70,66	1,36	245,22	4,99	52,33	125,85	6,75
7,00	3,60	14,00	1,22	114,75	29,51	25,71	32,00	68,34	1,65	224,43	4,32	59,17	152,72	10,20
7,00	3,50	13,60	1,08	125,93	32,41	25,74	39,23	76,97	1,63	239,58	4,37	48,42	152,68	12,18
