# Supplementary material for: Dose-dependent hemato-biochemical and genotoxic responses of common carp (Cyprinus carpio) to flupyradifurone
Source: Front Physiol. 2025 Oct 2;16:1676992. doi: 10.3389/fphys.2025.1676992 (PMC12528199; doi:10.3389/fphys.2025.1676992)
Supplement: Supplementary file 1 [file DataSheet1.zip › Peerj_Raw_Datas/head data.pdf]

	Group A	Group B	Group C	Group D	Group E	Group F	Group G
	Control	1 mg/L	3 mg/L	5 mg/L	25 mg/L	75 g/L	125 mg/L
1	19.470000	11.74000	12.18500	12.24000	11.41500	11.09500	10.63200
2	18.320000	11.83000	11.25600	11.98000	13.31000	10.62500	9.33100
3	19.130000	9.73000	9.23400	9.53000	11.94900	8.72400	9.45100
4	20.060000	11.65000	12.04900	10.51900	9.31500	10.24400	9.96200
5	17.460000	10.23000	12.57300	11.81600	10.74500	9.20200	11.87900
6	24.290000	11.62000	10.60400	9.02100	11.44800	8.29200	11.71400
7	23.570000	13.24000	11.38200	11.12900	12.88600	9.34700	10.70300
8	19.790000	10.00000	8.96200	8.94500	16.41600	10.44000	9.59300
9	16.810000	11.75000	9.20200	9.53800	14.75400	8.40900	10.16300
10	20.930000	10.45000	8.43600	8.59400	11.76500	12.83900	10.73800
11	17.880000	10.39000	11.57900	9.96200	14.41400	14.02400	10.01400
12	19.210000	9.18000	10.31000	9.30700	10.48300	11.12900	12.14200
13	20.680000	12.77000	9.48300	9.96900	8.70700	8.69900	11.42200
14	14.920000	11.58000	10.28100	10.32400	14.54900	12.06700	11.43500
15	24.200000	10.75000	9.24200	11.03400	10.20700	10.10400	7.17600
16	18.870000	9.09000	9.67100	10.51900	11.74000	12.39300	11.74000
17	22.580000	11.23000	11.68200	9.99200	8.26500	8.73300	9.93100
18	27.210000	10.24000	10.88400	12.48300	9.73300	13.34400	15.10600
19	20.250000	12.73000	10.18500	12.43500	9.90100	10.87000	8.93700
20	17.780000	12.11000	9.01200	11.27600	15.23500	13.24200	11.99900
21	22.200000	10.20000	10.66800	10.75900	10.09600	10.69600	10.60400
22	17.170000	13.36000	8.85300	11.30300	7.73000	12.24700	8.48000
23	18.680000	11.59000	7.35200	9.98400	10.39700	11.18300	9.53000
24	22.090000	11.24000	11.64300	10.03700	11.95500	11.69500	7.92200
25	17.880000	16.13000	10.94500	9.89400	14.83500	11.77800	10.60400
