# Supplementary material for: Dose-dependent hemato-biochemical and genotoxic responses of common carp (Cyprinus carpio) to flupyradifurone
Source: Front Physiol. 2025 Oct 2;16:1676992. doi: 10.3389/fphys.2025.1676992 (PMC12528199; doi:10.3389/fphys.2025.1676992)
Supplement: Supplementary file 1 [file DataSheet1.zip › Peerj_Raw_Datas/tail length data.pdf]

	Group A	Group B	Group C	Group D	Group E	Group F	Group G
	Control	1 mg/L	3 mg/L	5 mg/L	25 mg/L	75 g/L	125 mg/L
1	1.390714	7.07000	15.37750	19.46400	51.82550	39.40950	29.25900
2	1.308571	13.16500	15.64300	13.75800	51.96100	37.39950	34.98650
3	1.366429	10.47500	22.59900	13.64600	57.82650	48.05300	35.39850
4	1.432857	14.48500	21.36350	18.43850	38.55650	45.38600	32.09400
5	1.247143	9.68500	20.68350	34.33400	44.97350	44.67000	42.53450
6	1.735000	10.38000	20.52600	32.86650	42.35000	40.07200	39.06100
7	1.683571	5.72000	24.18000	17.15550	50.46600	45.97350	31.24950
8	1.413571	9.18000	21.96800	25.61250	53.65400	40.53000	34.53050
9	1.200714	9.38500	16.97500	16.38300	57.08000	50.14950	60.75650
10	1.495000	9.70500	13.29000	18.68000	53.05750	50.76850	48.65200
11	1.277143	9.32500	11.80950	14.11500	60.94100	60.53000	67.80000
12	1.372143	9.43000	13.66600	17.96850	47.97450	54.50450	63.29100
13	1.477143	13.42500	16.81150	13.92150	47.29650	47.98050	61.69600
14	1.065714	10.47000	15.62050	15.89300	51.53850	42.57150	64.49350
15	1.728571	7.73500	16.92300	17.02200	41.75950	46.23800	33.21900
16	1.347857	8.93500	14.65250	14.69350	59.40100	42.58150	33.66700
17	1.612857	8.25500	11.98200	15.04800	50.07050	51.48550	28.34950
18	1.943571	7.80000	21.29300	12.12050	48.49650	25.65100	25.66700
19	1.446429	9.71500	13.41650	12.97750	41.87450	30.68100	34.56450
20	1.270000	11.96500	26.83600	29.98600	55.92350	29.95000	40.31650
21	1.585714	13.64000	27.00200	20.06450	44.48800	35.01500	27.22600
22	1.226429	9.13000	18.10150	28.39350	45.09900	27.08250	33.41000
23	1.334286	12.20500	15.45000	14.84400	54.93750	32.28050	20.88900
24	1.577857	7.92000	7.88850	14.87250	57.97650	33.93550	29.82300
25	1.277143	5.21500	17.47950	28.59100	52.35850	32.53200	27.29300
