# Supplementary material for: Dose-dependent hemato-biochemical and genotoxic responses of common carp (Cyprinus carpio) to flupyradifurone
Source: Front Physiol. 2025 Oct 2;16:1676992. doi: 10.3389/fphys.2025.1676992 (PMC12528199; doi:10.3389/fphys.2025.1676992)
Supplement: Supplementary file 1 [file DataSheet1.zip › Peerj_Raw_Datas/tail moment length data.pdf]

	Group A	Group B	Group C	Group D	Group E	Group F	Group G
	Control	1 mg/L	3 mg/L	5 mg/L	25 mg/L	75 g/L	125 mg/L
1	6.490000	9.40500	13.78125	15.85200	31.62025	25.25225	19.94550
2	6.106667	12.49750	13.44950	12.86900	32.63550	24.01225	22.15875
3	6.376667	10.10250	15.91650	11.58800	34.88775	28.38850	22.42475
4	6.686667	13.06750	16.70625	14.47875	23.93575	27.81500	21.02800
5	5.820000	9.95750	16.62825	23.07500	27.85925	26.93600	27.20675
6	8.096667	11.00000	15.56500	20.94375	26.89900	24.18200	25.38750
7	7.856667	9.48000	17.78100	14.14225	31.67600	27.66025	20.97625
8	6.596667	9.59000	15.46500	17.27875	35.03500	25.48500	22.06175
9	5.603333	10.56750	13.08850	12.96050	35.91700	29.27925	35.45975
10	6.976667	10.07750	10.86300	13.63700	32.41125	31.80375	29.69500
11	5.960000	9.85750	11.69425	12.03850	37.67750	37.27700	38.90700
12	6.403333	9.30500	11.98800	13.63775	29.22875	32.81675	37.71650
13	6.893333	13.09750	13.14725	11.94525	28.00175	28.33975	36.55900
14	4.973333	11.02500	12.95075	13.10850	33.04375	27.31925	37.96425
15	8.066667	9.24250	13.08250	14.02800	25.98325	28.17100	20.19750
16	6.290000	9.01250	12.16175	12.60625	35.57050	27.48725	22.70350
17	7.526667	9.74250	11.83200	12.52000	29.16775	30.10925	19.14025
18	9.070000	9.02000	16.08850	12.30175	29.11475	19.49750	20.38650
19	6.750000	11.22250	11.80075	12.70625	25.88775	20.77550	21.75075
20	5.926667	12.03750	17.92400	20.63100	35.57925	21.59600	26.15775
21	7.400000	11.92000	18.83500	15.41175	27.29200	22.85550	18.91500
22	5.723333	11.24500	13.47725	19.84825	26.41450	19.66475	20.94500
23	6.226667	11.89750	11.40100	12.41400	32.66725	21.73175	15.20950
24	7.363333	9.58000	9.76575	12.45475	34.96575	22.81525	18.87250
25	5.960000	10.67250	14.21225	19.24250	33.59675	22.15500	18.94850
